# Prevalence and correlates of needle-stick injuries among active duty police officers in Tijuana, Mexico

**DOI:** 10.7448/IAS.19.4.20874

**Published:** 2016-07-18

**Authors:** María Luisa Mittal, Leo Beletsky, Efraín Patiño, Daniela Abramovitz, Teresita Rocha, Jaime Arredondo, Arnulfo Bañuelos, Gudelia Rangel, Steffanie A Strathdee

**Affiliations:** 1 Division of Global Public Health, Department of Medicine, University of California San Diego, La Jolla, CA, USA; 2 School of Medicine, Universidad Xochicalco, Tijuana, México; 3 School of Law & Bouvé College of Health Sciences, Northeastern University, Boston, MA, USA; 4 Public Safety Support Department, Dirección Municipal de Salud, Tijuana, México; 5 Department of Planning and Special Projects, Secretaría de Seguridad Pública Municipal, Tijuana, México; 6 Comisión de Salud Fronteriza México-EEUU, Sección México, Tijuana, México; 7 Department of Migrant Health, Secretaría de Salud, México DF, México

**Keywords:** occupational accidents, HIV, viral hepatitis, syringe disposal, syringe confiscation, policing, law enforcement, harm reduction

## Abstract

**Introduction:**

Police officers are at an elevated risk for needle-stick injuries (NSI), which pose a serious and costly occupational health risk for HIV and viral hepatitis. However, research on NSIs among police officers is limited, especially in low- and middle-income countries. Despite the legality of syringe possession in Mexico, half of people who inject drugs (PWID) in Tijuana report extrajudicial syringe-related arrests and confiscation by police, which has been associated with needle-sharing and HIV infection. We assessed the prevalence and correlates of NSIs among Tijuana police officers to inform efforts to improve occupational safety and simultaneously reduce HIV risks among police and PWID.

**Methods:**

Tijuana's Department of Municipal Public Safety (SSPM) is among Mexico's largest. Our binational, multi-sectoral team analyzed de-identified data from SSPM's 2014 anonymous self-administered occupational health survey. The prevalence of NSI and syringe disposal practices was determined. Logistic regression with robust variance estimation via generalized estimating equations identified factors associated with ever having an occupational NSI.

**Results:**

Approximately one-quarter of the Tijuana police force was given the occupational health survey (*N*=503). Respondents were predominantly male (86.5%) and ≤35 years old (42.6%). Nearly one in six officers reported ever having a NSI while working at SSPM (15.3%), of whom 14.3% reported a NSI within the past year. Most participants reported encountering needles/syringes while on duty (*n*=473, 94%); factors independently associated with elevated odds of NSIs included frequently finding syringes that contain drugs (adjusted odds ratio (AOR): 2.98; 95% confidence interval (CI): 1.56–5.67) and breaking used needles (AOR: 2.25; 95% CI: 1.29–3.91), while protective factors included being willing to contact emergency services in case of NSIs (AOR: 0.39; 95% CI: 0.22–0.69), and wearing needle-stick resistant gloves (AOR: 0.43; 95% CI: 0.19–0.91).

**Conclusions:**

Tijuana police face an elevated and unaddressed occupational NSI burden associated with unsafe syringe-handling practices, exposing them to substantial risk of HIV and other blood-borne infections. These findings spurred the development and tailoring of training to reduce NSI by modifying officer knowledge, attitudes and enforcement practices (e.g. syringe confiscation) – factors that also impact HIV transmission among PWID and other members of the community.

## Introduction

Syringe confiscation and syringe-related arrests by police are widespread in settings where injection drug use is prevalent [1–11]. Handling used syringes in high-risk areas can expose law enforcement personnel to a serious occupational health risk [12–29]. Occupational needle-stick injuries (NSIs) carry a 0.2 to 0.5% risk of HIV acquisition, while the odds of acquiring viral hepatitis are substantially higher (3–10% for hepatitis C virus (HCV) and 2–40% for hepatitis B virus (HBV)) [30]. Documented virus survival lasting more than four weeks for HIV and more than two months for HCV in contaminated syringes contribute to the hazards of occupational exposure [15,19,31]. Additionally, police officers also report substantial anxiety about NSI risk, fear of HIV disease transmission and a burden on family relationships, contributing to the alarmingly elevated levels of job-related stress and turnover among police [2,20,32]. Physical and mental health harms aside, financial and human resource costs of NSIs can be very high [33].

Police officers have some of the greatest occupational exposures to sharps (objects that may lacerate or puncture skin) as well as blood and bodily fluids [2,17,18,21–28,34]. The New York City Police Department has reported a transcutaneous exposure rate of 38.7 per 10,000 officers per year [24]. In Denver, 9.5% of occupational exposures to blood were from NSIs [18]. In the 1990s, 0.9% of police officers in Atlanta, Fulton and Oakland, reported NSIs via used syringes in the past six months [22]. In Amsterdam, 11% of police officers reported an occupational NSI during a four-year period [25]. The lifetime prevalence of reported occupational NSIs among police officers varies widely (e.g. 3.8% in North Carolina,6.4% in Rhode Island, 29.7% in San Diego, California, in the United States), and is thought to be greatly under-reported [2,26,27]. Yet, there is little research focusing on this occupational hazard and its prevention.

Situated along a major drug trafficking route, Tijuana, Mexico, exhibits elevated levels of injection drug use, along with concentrated epidemics of HIV and viral hepatitis: 3.0% HIV, 85% HBV and 96 to 98.7% HCV [35,36]. Police occupational risk from NSI in this locale may be compounded by risk of infection from the number of high dead-space syringes, popular in Mexico, due to blood retained in the syringe hubs [37–39]. Despite the legality of syringe possession and the decriminalization of small-scale drug possession in August 2009, 48 to 57% of Tijuana's people who inject drugs (PWID) report syringe-related arrests and confiscation by police [3–5,40]. As elsewhere globally, such encounters have been associated with HIV risk behaviours and HIV acquisition among PWID [3–5].

In previous literature, a number of NSI risk factors have been identified among law enforcement, including working for less than 10 years, evening shifts, pat-down searches, little or no time to put on protective gloves, lack of needle-stick resistant gloves and serving on street-level patrol [18,24,26]. Adverse interactions with PWID, including harassment and syringe confiscation in HIV incidence hotspots, can also increase NSI risk for police, especially since PWID may not be willing to volunteer syringe possession during searches [23,41]. As part of a larger effort to align policing and public health efforts to prevent the spread of blood-borne pathogens in Tijuana, Mexico, we assessed the prevalence and correlates of NSIs among police officers, hypothesizing that officers who engage in syringe confiscation and syringe-related arrests may be at an elevated risk of NSI.

## Methods

### Study population

The *Secretaría de Seguridad Pública Municipal* (SSPM, Department of Municipal Public Safety) in Tijuana is among Mexico's largest municipal police force with approximately 2100 active duty officers. Approximately 80% of the force is male, the mean age is 38 years old, about 60% have completed high school and/or higher education, 56.6% report a monthly of income of less than 15,000 pesos (around $1000 USD) and 95.3% report access to universal healthcare services [42]. Officers must be at least 18 years old and, since 2009, are required to have a high school diploma when entering the force [43].

### Survey development

An anonymous self-administered survey of a convenience sample of active duty police officers was conducted during June and July 2014. The instrument covered socio-demographics, work environment and occupational health domains. Occupational health survey items focused on safety practices in the line of duty (e.g. syringe encounters and handling), occupational accidents (e.g. “Have you ever had any NSIs while you were working?”) and post-exposure follow-up (e.g. seeking medical attention).

### Data collection

Data was collected by SSPM throughout 11 police districts and three special departments (i.e. tourist, special ops and forensic/expert police). The police officers who participated in the study completed self-administered surveys while on duty. Surveys were completed in a closed room within the precinct, lasting approximately 15 minutes. Existing data without person-identifiable information was approved by SSPM for a secondary analysis by the UCSD research team through a Memorandum of Understanding. We obtained IRB exemption for analysis of de-identified data from the UC San Diego Human Research Protections Program.

### Statistical analysis

Chi-Square tests were used to compare police officers who reported ever having sustained a NSI versus those who did not. To identify individual factors associated with having at least one NSI, univariate and multivariate logistic regression models used generalized estimating equations with robust variance estimation [44]; variables that yielded in the univariate regressions *p*-values equal or less than 0.10 were considered for inclusion in the multivariable model. The model was checked for integrity by examining and ruling out interactions and multi-collinearity. Analyses were performed using SPSS Statistics Version 21 (IBM, Armonk, New York).

## Results

### Socio-demographics and occupational characteristics

A total of 531 active duty police officers participated, representing approximately one-quarter of the entire Tijuana police force. Most (*N*=503; 95%) answered the occupational health portion of the survey. The surveyed officers were predominantly male 86.5%, 13.1% female; more than half were older than 35 years (see Table 1). Surveyed officers have lived almost 30 years in Tijuana on average and have worked in the police force for an average almost 10 years. The most frequent rank was officer (92.4%), with 84.5% reported patrolling in sedans or vans as their main duty.

**Table 1:** Socio-demographic and work environment characteristics of Tijuana police officers (*N*=503)

Variable	Ever experienced NSI, *n*=77	Did not experience NSI, *n*=426	*p*	Total, *N*=503
Socio-demographic characteristics				
Male	65 (84.4%)	370 (86.9%)	0.45	435 (86.5%)
Female	12 (15.6%)	54 (12.7%)	0.45	66 (13.1%)
Age ≤35 years old	34 (44.2%)	180 (42.3%)	0.78	214 (42.5%)
Age >35 years old	43 (55.8%)	246 (57.7%)	0.77	289 (57.5%)
Mean years lived in Tijuana	27.75	27.33	0.79	27.39
Work environment				
Mean years worked at SSPM	11.16	9.59	0.02	9.38
Job position: police officer	70 (90.9%)	368 (86.4%)	0.44	438 (87.0%)
Main duty patrol (sedan/van)	61 (79.2%)	338 (79.3%)	0.98	399 (79.3%)
Occupational safety in the line of duty				
Ever encounters syringes when searching a person	76 (98.7%)	397 (93.2%)	0.06	473 (94.0%)
Ever encounters syringes that contain drugs when searching a person	70 (90.9%)	364 (85.4%)	0.20	434 (86.3%)
Rarely encounters new/unused syringes when searching a person	33 (42.9%)	157 (36.9%)	0.32	190 (37.8%)
Used syringe disposal, *n*=423				
Biohazard sharps container	2 (2.6%)	23 (5.4%)	0.25	25 (5.9%)
Other resistant receptacle	6 (7.8%)	36 (8.5%)	0.71	42 (9.9%)
Throw in sewer or ditch	3 (3.9%)	18 (4.2%)	0.78	21 (5.0%)
Break them	35 (45.5%)	109 (25.6%)	0.00	144 (34.0%)
Throws in trash	24 (31.2%)	128 (30.0%)	0.84	152 (36.0%)
Asks person to throw in trash	12 (15.6%)	56 (13.1%)	0.57	68 (16.1%)
Retain to present to authorities	12 (15.6%)	79 (18.5%)	0.36	91 (21.5%)
Occupational accidents				
NSI was in past year, *n*=77	11 (14.3%)	–	–	11 (14.3%)
Would report NSI to supervisor	13 (16.9%)	58 (13.6%)	0.46	71 (14.1%)
Aware of standard NSI response protocol	21 (27.3%)	98 (23.0%)	0.45	119 (23.7%)
Post-exposure experience				
Ever sought medical attention post NSI, *n*=77	51 (66.2%)	–	–	51 (66.2%)
Reasons for not reporting NSI, *n*=62				
Did not have time to report it	7 (11.3%)	–	–	7 (11.3%)
Did not know what was the procedure to report it	13 (21.0%)	–	–	13 (21.0%)
I didn't think it was important to report	14 (22.6%)	–	–	14 (22.6%)

NSI: needle-stick injury; SSPM: Secretaría de Seguridad Pública Municipal (Tijuana Police Department).

The overwhelming majority (94%) of officers reported ever encountering syringes while on duty. Almost 40% of all surveyed officers said it was rare to find new syringes when searching a person. When searching a person, 86.3% of officers reported ever encountering syringes that contain drugs. When asked what they did when encountering syringes that contain drugs, more than half said they put them in the trash. Alarmingly, one-third reported breaking syringes and more than one-fifth said they retained them to present to the authorities. Overall, a troublingly low number of officers (5.9%) reported proper syringe disposal (see Table 1).

### Prevalence of NSIs

Of the 503 police officers that answered the occupational accidents portion of the questionnaire, 77 (15.3%) reported ever having a NSI during their work at SSPM, of whom 11 (14.3%) reported a NSI in the last year. Only two-thirds of officers (66.2%) sought medical attention after having a NSI. About one-fifth of officers who had a NSI said they did not think it was important to report NSIs (22.6%) (see Table 1).

### Correlates of NSIs

Univariate analysis identified the number of years working for SSPM, frequently encountering syringes, using no measures to prevent NSIs, and breaking syringes that contain drugs as factors associated with having at least one NSI (see Table 2).

**Table 2:** Correlates of lifetime needle-stick injuries among Tijuana police officers

	*n*^a^	Unadjusted odds ratio (95% CI)
Socio-demographic characteristics		
35 years old or younger	503	1.08 (0.66–1.76)
Number of years lived in Tijuana	498	1.00 (0.98–1.03)
Work environment		
Number of years working at SSPM	503	1.03 (1.00–1.06)*
Worked for at least 1 year at SSPM	503	2.77 (0.84–9.16)
Worked for at least 5 years at SSPM	424	3.03 (0.91–10.07)
Foot patrol as main duty	473	1.81 (0.78–4.19)
Occupational safety in the line of duty		
Encounters syringes frequently or all the time	499	2.16 (1.29–3.62)*
Encounters syringes that contain drugs frequently or all the time	479	2.34 (1.29–4.26)*
Encounters injection related drug paraphernalia frequently or all the time	476	1.95 (1.121–3.39)
Syringe disposal when encountering syringes that contain drugs		
Put them in biohazard receptacle for sharp objects	423	0.43 (0.10–1.87)
Put them in other resistant receptacle (i.e. detergent bottle)	423	0.84 (0.34–2.08)
Package them to present to authorities	423	0.73 (0.38–1.43)
Occupational accidents		
Measures to prevent NSIs		
Wear latex gloves	486	1.08 (0.62–1.87)
Wears needle-resistant gloves	486	0.48 (0.25–0.95)*
Ask suspect to-be-registered if they have sharp objects	486	0.83 (0.50–1.39)
Only handle capped syringes	486	0.60 (0.08–4.84)
Deposit confiscated syringes into receptacle	486	0.53 (0.16–1.78)
None	486	2.06 (1.04–4.08)*
In case of experiencing a NSI		
Would contact immediate supervisor	487	1.28 (0.66–2.47)
Would not contact anybody	487	2.52 (0.76–8.41)
Would contact emergency services	487	0.49 (0.30–0.82)*
Would not know who to contact	487	2.52 (0.76–8.41)
Aware of standard NSI response protocol	485	1.24 (0.71–2.15)
When encountering syringes that contain drugs		
Asks suspect to put syringe in trash	423	1.12 (0.57–2.22)
Throws syringe into the trash	423	0.94 (0.55–1.62)
Breaks syringes that contain drugs	423	2.31 (1.37–3.90)*
Throws them in sewer or ditch	423	0.85 (0.24–2.96)

a Change in sample size due to different number of observations available for each variable. 95% CI: 95% confidence intervals

* significant at *p*<0.05; SSPM: Secretaría de Seguridad Pública Municipal (Tijuana Police Department); NSI: needle-stick injury.

Among the officers who reported ever encountering needles or syringes that contain drugs while on duty (*n*=434), factors independently associated with having NSIs included frequently finding syringes that contain drugs while on duty (adjusted odds ratio (AOR): 2.98; 95% confidence interval (CI): 1.56–5.67), breaking used needles when encountering them (AOR: 2.25; 95% CI: 1.29–3.91), in case of NSI contacting emergency services (AOR: 0.39; 95% CI: 0.22–0.69), wearing needle-stick resistant gloves as a measure to prevent NSIs (AOR: 0.43; 95% CI: 0.19–0.91) (see Table 3).

**Table 3:** Factors independently associated with experiencing any lifetime needle-stick injuries among Tijuana police officers who encountered syringes that contain drugs while on duty (*n*=434^a^)

	Adjusted odds ratio	95% confidence interval*
Number of years in the department	1.04	1.01–1.08
Encounters syringes that contain drugs frequently or all the time	2.98	1.56–5.67
Breaks syringes that contain drugs	2.24	1.29–3.90
Wears needle-resistant gloves	0.43	0.19–0.91
Would contact emergency services in case of NSI	0.39	0.22–0.69

a Due to missing data (as noted in Table 2), only 407 observations were used in the model.

* All significant at *p*<0.05. NSI: needle-stick injury.

## Discussion

This study of active duty police officers in Tijuana found that nearly one in six officers reported ever having an occupational NSI, of whom a similar proportion reported having had a NSI in the last year. These findings are especially salient in view of alarming levels of very risky syringe-handling practices – all in an environment in which PWID have a higher prevalence of blood-borne viral infections than the rest of the population. Lifetime prevalence of NSI among our respondents was four times greater than in a recent North Carolina study and more than twice the prevalence in Rhode Island; however, it was half the reported prevalence in the adjacent border city of San Diego, California [2,26,27]. Overall, the prevalence of NSI in our and other samples may be under-reported, as only two out of three officers (66.2%) reported seeking medical attention post-exposure. Nevertheless, the proportion seeking medical attention was higher as compared to other settings. This study did not present an opportunity to ascertain whether experiencing a NSI resulted in actual disease transmission or HIV post-exposure prophylaxis (HIV-PEP) [24–26]. In other settings, 8 to 19% of officers who report a NSI start HIV-PEP [17,21,25], but the SSPM did not have an occupational NSI response or PEP programme when this study was conducted. Lack of uniform measures of NSI time periods across different research studies (i.e. in the past six months, past year, four-year period or lifetime) and inconsistency in the type of transcutaneous exposure (i.e. NSI, human bite or other sharp object) do not allow for more precise cross-sample comparisons [2,24–28].

The confluence of poor implementation of drug policy provisions and poor occupational safety knowledge and practices creates an overall risk environment that endangers both police and PWID. The overwhelming majority of our respondents encountered syringes that contain drugs in their daily duties, which was independently associated with three times higher odds of NSI. Recent changes in drug laws (i.e. the decriminalization of small-scale drug possession) should precipitate the discontinuation of syringe confiscation, but our other research suggests that this law – as well as the long-standing legal status of syringes – may be often ignored in day-to-day drug law enforcement practices [4]. As a result, police continue to put themselves at risk of NSI by handling syringes during encounters and undertake transporting them to legal authorities for weighing [4]. Our data supplements previous research on street-level policing behaviours (e.g. syringe confiscation and disposal) that, in addition to elevating police occupational risk, also led PWID to share syringes and experience elevated HIV risk or overdose mortality [3–6,45]. These policing practices contribute to the global HIV pandemic by increasing drug-related behaviours among PWID and placing police officers at higher risk for NSI [6–9,46].

Our findings also provide a baseline prevalence of protective behaviours that offer a promising platform for intervention. Our analysis suggests that police officers wearing needle-stick resistant gloves had lower odds of NSI. Needle-stick resistant gloves offer a protective barrier for officers exposed to syringes but are not provided by Mexican police departments. With more than half of police officers earning $15,000 Mexican pesos per month (~$938 USD) [42], purchasing these gloves in Mexico can amount to 10% of their monthly household income. Anecdotally, some officers disliked using these gloves because they reduced sensitivity during pat-down searches.

Our analysis found that officers who break syringes had two times higher odds of experiencing a NSI. Although this finding is not unexpected, to our knowledge, this is the first time this alarming behaviour has been systematically documented among police officers in association with NSI risk. Fortunately, this behaviour could be reversed: studies have shown that police officers are receptive to occupational safety techniques, including safe syringe disposal [2,34]. Interestingly, for officers frequently encountering syringes in their daily activities, considering contacting emergency services in case of NSI was a protective factor for NSI. Decreased NSI-related anxiety, based on improved knowledge of proper NSI protocols, could reduce unjustified syringe confiscation, leading to both occupational and public health benefits [2,10].

When our binational, multi-sectoral team presented these data to law enforcement and municipal authorities in Tijuana in 2014, the mayor requested that our team work with the SSPM to implement a Police Education Program (PEP) focused on occupational safety and HIV prevention that was mandated to all active duty police officers [29]. The resulting programme, which is being implemented and evaluated through *Proyecto ESCUDO Tijuana* (Project SHIELD), is taught by police academy instructors and addresses occupational safety transmission, prevention and treatment of HIV and related infections (sexually transmitted infections and viral hepatitis), safe syringe disposal and HIV-PEP [29]. Syringe and drug possession laws are also addressed in order to reduce occupational risk [2,29,34]. In parallel, we also introduced a NSI response and surveillance system for the entire force. This project strengthened the relationship between public health and public safety and the creation of a system-wide NSI surveillance programme that can be used for further studies necessary to determine the quality and time-sensitive access and implementation of programmes to help increase preventative measures such as hepatitis B immunization [19–22,24,29]. Increasing PWID access to low dead-space syringes and aligning harm reduction efforts with law enforcement practices may further diminish the HIV and viral hepatitis risks of both police officers and the broader community [9,11,38].

Our study limitations included limited generalizability due to convenience sampling and a likely underestimate of reported frequencies of NSI due to self-reporting [18,26]. Under-reporting of NSIs has been found in other settings due to lack of awareness of requirements and policies, lack of time, perception that injuries were low risk, fear of outcome and dissatisfaction with follow-up procedures [16]. Survey administration by SSPM peers while on duty may have also contributed to under-reporting.

Despite these limitations, this is the first study of its kind among police officers in Latin America, and to our knowledge, among the first to examine NSIs among police in low- and middle-income countries. Our findings led to a unique binational and multi-sectoral collaboration addressing public health and public safety concerns. For police officers, handling used syringes is a serious occupational and healthcare risk. Knowledge of this occupational health risk can provide sufficient motivations for officers to change policing behaviours while protecting their health [2,47].

## Conclusions

Police officers face a substantial, but poorly addressed risk of occupational risk from NSI. This study highlights the need for evidence-driven prevention, including training to help police officers reduce their risk of NSIs while modifying policing behaviours to improve the HIV risk environment for PWID [29]. These data also helped support the need for a standardized system to track exposures and injuries that may facilitate urgent access to integral healthcare services including HIV-PEP. Because syringe and small-scale drug possession is legal in Mexico, law enforcement authorities should discontinue syringe confiscation practices in order to decrease unnecessary occupational exposures while preventing PWID from sharing syringes.
